# New Era in Plant Alternative Splicing Analysis Enabled by Advances in High-Throughput Sequencing (HTS) Technologies

**DOI:** 10.3389/fpls.2019.00740

**Published:** 2019-06-04

**Authors:** Renesh Bedre, Sonia Irigoyen, Ezequiel Petrillo, Kranthi K. Mandadi

**Affiliations:** ^1^Texas A&M AgriLife Research and Extension Center, Texas A&M University, Weslaco, TX, United States; ^2^Departamento de Fisiología, Biología Molecular y Celular, Facultad de Ciencias Exactas y Naturales, Universidad de Buenos Aires, Buenos Aires, Argentina; ^3^CONICET-Universidad de Buenos Aires, Instituto de Fisiología, Biología Molecular y Neurociencias (IFIBYNE), Buenos Aires, Argentina; ^4^Department of Plant Pathology and Microbiology, Texas A&M University, College Station, TX, United States

**Keywords:** alternative splicing, high-throughput sequencing, bioinformatics, RNA-seq, PCR, non-sense-mediated decay

Alternative splicing (AS) is a crucial posttranscriptional mechanism of gene expression which promotes transcriptome and proteome diversity. At the molecular level, splicing and AS involves recognition and elimination of intronic regions of a precursor messenger RNA (pre-mRNA) and joining of exonic regions to generate the mature mRNA. AS generates more than one mRNA transcript (transcripts) differing in coding and/or untranslated regions (UTRs). AS can be classified into four major types including the exon skipping (ES), intron retention (IR), alternative donor (AD), and alternative acceptor (AA), of which IR is the most prevalent event in plants (Mandadi and Scholthof, 2015). In addition to these AS types, a subfamily of IR called exitrons, which has dual features of introns and protein-coding exons were first reported in *Arabidopsis thaliana (Arabidopsis)* and later also found in humans (Marquez et al., 2015). These spliced transcripts influence multiple biological processes such as growth, development and response to biotic and abiotic stresses in plants (Filichkin et al., 2015; Mandadi and Scholthof, 2015; Wang et al., 2018a).

## Functional Relevance of AS

AS can produce aberrant or unstable transcripts with premature termination codons (PTCs). The PTC-containing transcripts are often targeted to degradation by a conserved cytoplasmic RNA degradation mechanism called NMD (non-sense-mediated mRNA decay). NMD mechanisms ensure that there is a balance or homeostasis in the functional vs. non-functional transcripts (Kalyna et al., 2012). In contrast to mammals, where NMD targets are degraded by a suppressor with morphogenetic effect on genitalia (SMG7) endonucleolytic pathway, plants NMD primarily occurs via. SMG7 exonucleolytic pathway (Shaul, 2015). AS can also produce stable transcripts, which encode proteins with altered functional domains, subcellular localization, and/or biological functions (Reddy et al., 2013; Shang et al., 2017). In humans, ~15% of genetic diseases are a result of aberrant splicing (Staiger and Brown, 2013). In plants, several studies have showed that AS has biologically significant implications in growth, development, stress-responses, and/or adaptation. For instance, AS in a MADS-box transcription factor gene, *SHORT VEGETATIVE PHASE (SVP)*, results in multiple transcripts (*SVP1* and *SVP3*), which encode proteins with altered interaction domains (Severing et al., 2012). Overexpression of *SVP1*, but not *SVP3*, resulted in repression of flowering (Severing et al., 2012). In rice, AS occurs in the *DEHYDRATION-RESPONSIVE ELEMENT BINDING PROTE IN 2 (DREB2B)* gene, but only when subjected to drought and heat stress, and results in the production of an alternative transcript which encodes the full-length functional protein that confers tolerance to the stresses (Matsukura et al., 2010). Similarly, in tobacco, the classical resistance gene (*N*) against *Tobacco mosaic virus* (TMV) is alternatively spliced, resulting in production of two forms—a short and a long transcript (Dinesh-Kumar and Baker, 2000). Functional analysis revealed that both transcripts are required in certain ratio to confer full resistance to TMV (Dinesh-Kumar and Baker, 2000). Recently, by employing high-throughput sequencing (HTS), Mandadi and Scholthof (2015), identified ~670 intron-containing genes in Brachypodium that were aberrantly spliced in response to viral infection. Several of these genes encoded resistance proteins, transcription factors, and splicing factors (Mandadi and Scholthof, 2015). Together, these studies suggest that many AS events, if not all, have biologically-significant implications in plant growth, development and response to stresses.

## High-Throughput Sequencing (HTS) for AS Analysis

Historically, our knowledge of plant alternative splicing and how it affects biological processes was primarily gleaned from studies of few plant species (e.g., *Arabidopsis*, rice; Modrek and Lee, 2002). However, with the rapid developments in HTS (a.k.a. next- and third-generation sequencing) technologies, particularly long-read, single-molecule real-time sequencing (SMRT) and direct RNA-sequencing platforms, the field is rapidly changing. Several existing and emerging next- generation sequencing (NGS) platforms, and bioinformatics tools are useful for genome-wide queries of AS in diverse plant species (Filichkin et al., 2010; Mandadi and Scholthof, 2015; Thatcher et al., 2016). HTS-based genome analysis studies estimated that ~33–70% of plant genes undergo AS, suggesting a broader influence of AS in shaping the functional transcriptome and proteome landscapes of plants (Pan et al., 2008; Chamala et al., 2015; Filichkin et al., 2015; Mandadi and Scholthof, 2015; Wang et al., 2018a). The seemingly lower number of genes undergoing AS in plants when compared to humans (~95%) could be due to lack of enough studies or in-depth annotations of the plant genomes. In early reports dating back to 2004, the AS rates in the model plant *Arabidopsis* was reported at a meager ~11.6%, when the AS rates in humans was ~42% (Iida et al., 2004). Efforts by several groups over the years, and with the advent of HTS technologies, the AS rates in *Arabidopsis* and humans ascended comparably to ~60 and ~95%, respectively (Wang and Brendel, 2006; Zhang et al., 2015; Laloum et al., 2018). Hence, we presume that with recent advances in HTS technologies, the AS frequencies in plant species would likely increase further. Alternatively, it is quite possible that differential gene structure/number, spliceosome composition, as well as variations in the types of tissues sampled, and detection methods could contribute to the observed lower AS rate in plants when compared to humans.

**Figure 1:**
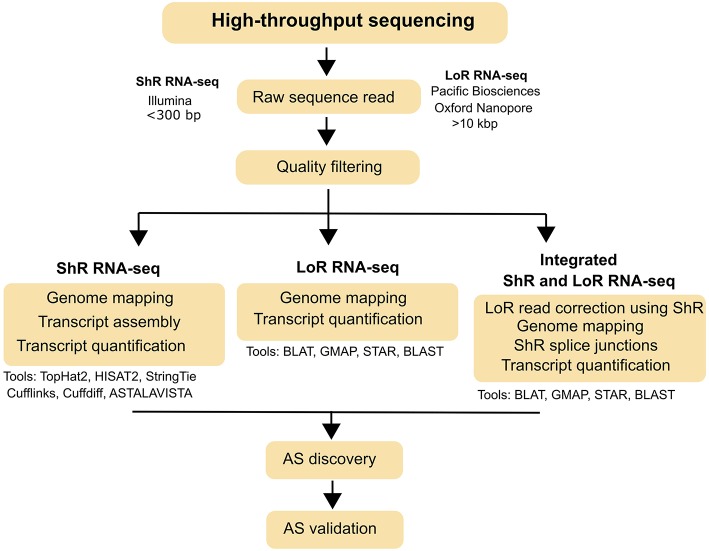
Typical workflow for AS identification from short read (ShR) and long read (LoR) RNA-seq. AS identification strategies using ShR and LoR RNA-seq and the various bioinformatics tools that can be used for each strategy are also indicated.

HTS-based short read (Illumina) and long read (Pacific Biosciences and Oxford Nanopore) sequencing technologies have revolutionized the field of DNA and RNA sequencing. Specifically, short read (<300 bp) RNA-sequencing (ShR RNA-seq), which integrates qualitative (gene discovery) and quantitative (gene quantification) assays, became a popular tool for genome-wide AS identification in plants as well as in other organisms. Because ShR RNA-seq provides high sequencing depth, a low error rate (<1%) and relatively-lower cost, it has been extensively used to characterize and quantify spliced transcripts in well-annotated plant genomes such as *Arabidopsis* (Calixto et al., 2018), *Oryza sativa* (Zhang and Xiao, 2018), *Brachypodium distachyon* (Mandadi and Scholthof, 2015), *Zea mays* (Thatcher et al., 2016), and *Glycine max* (Shen et al., 2014). Further, discovery of AS in plants was improved by the continuous development of open-source bioinformatics tools and pipelines. Identifying spliced transcripts from ShR-RNA-seq involves, mapping of high quality reads to reference genomes (HISAT2, TopHat2), transcript assembly (StringTie, Cufflinks, Trinity), AS events analysis (ASTALAVISTA), and transcript quantification (Cuffdiff, DESeq2) (Figure 1; Haas et al., 2013; Trapnell et al., 2013; Love et al., 2014; Foissac and Sammeth, 2015; Pertea et al., 2016; Irigoyen et al., 2018). Among these tools, HISAT2 and StringTie analysis pipeline (new Tuxedo package; Pertea et al., 2016) perform much faster, requires less memory and generates more accurate results over the TopHat2 and Cufflinks analysis pipeline (original Tuxedo package; Trapnell et al., 2012).

Despite the advances in ShR RNA-seq for AS discovery, this technology has limited scope in polyploids plant species (e.g., sugarcane, cotton) without reference genomes or those lacking comprehensive transcript-level annotations. To overcome this limitation, long read RNA-sequencing (LoR RNA-seq) technologies such as Pacific Biosciences (SMRT sequencing) and Oxford Nanopore (MinION) which has the ability to sequence full-length transcripts and direct RNA sequencing, have been used to study complex AS landscapes in polyploids (Liu et al., 2017; Wang et al., 2018a). LoR RNA-seq using SMRT (Iso-Seq method) and MinION can generate exceptionally long reads (>10 Kbp), which could cover most of the full-length eukaryotic transcripts and thus eliminate the need for transcript assembly (Liu et al., 2017; Ardui et al., 2018). Bioinformatics steps to identify AS from LoR RNA-seq involves mapping of high quality reads to reference genome (GMAP, BLAT) and, identifications of alternative transcripts and AS events from the alignments (Figure 1; Kent, 2002; Wu and Watanabe, 2005). For species where a reference genome is not available, the self-BLAST based pipeline on LoR RNA-seq can be used to detect AS based on the INDELs (Figure 1; Liu et al., 2017). Even though LoR RNA-seq is exceptional in resolving transcript structures, it has some limitations when compared to ShR RNA-seq including lower sequencing depth, high error-rate (up to ~15%), and being poorly suited for transcript quantifications (Ardui et al., 2018; Clark et al., 2018). These limitations in LoR RNA-seq could be mitigated by employing an integrated strategy involving both LoR and ShR RNA-seq reads (Figure 1; Koren et al., 2012). However, error correction methods are highly dependent on the availability of reference genomes and transcript annotations (Liu et al., 2017).

## Validation of AS

Before embarking on functional analyses, it is recommended to validate the AS events using conventional molecular biology techniques. Reverse transcription followed by polymerase chain reaction (RT-PCR), cloning and Sanger-based sequencing are widely used approaches to confirm the presence and the sequence of the alternatively spliced transcripts (Simpson et al., 2008, 2016; Mandadi and Scholthof, 2015). Semi-quantitative RT-PCR (SqRT-PCR) and quantitative RT-PCR (qRT-PCR) can be used to quantify the alternatively spliced transcripts to understand AS regulation in different conditions (Harvey and Cheng, 2016). Both methods require cDNA synthesis as a first step, using either oligo-dT, which amplifies several mRNAs from the same sample, or specific oligos to generate cDNAs from specific transcripts. SqRT-PCR estimates relative amounts of the different templates in a sample and can be used to compare changes across different conditions/treatments. Additionally, to quantify the relative expression of the transcripts, quantitative PCR (qPCR) could be performed. In qPCR-based assays, transcript-specific primers are designed to quantify the expression levels. Subsequent to a PCR-based analysis, the various transcripts can be resolved in an agarose gel and purified for further cloning and Sanger-based sequencing to validate the sequence. Although less frequently employed, other molecular-biology methods that do not require PCR such as the Northern blotting and RNAse protection assays, can also be utilized to validate alternatively spliced transcripts, particularly when the size differences between transcripts allow a clear distinction. Furthermore, large-scale proteomics experiments (e.g., mass spectrometry) can be used to identify and study the proteins resulting from the alternatively-spliced transcripts and to support the *in-silico* protein sequence predictions (Tress et al., 2017).

Lastly, the AS analysis combined with functional genetic experiments will ultimately allow understanding of the biological significance of the various alternatively spliced transcripts. These methods typically involve selective overexpression or knockdown of the transcripts (and the encoded proteins) using stable or transient plant transformations, followed by evaluation of the trait of interest. Biochemical experiments to decipher the encoded protein localization, protein-protein, and protein-RNA interactions can also provide mechanistic insights into the functions of the alternatively spliced transcripts (Dinesh-Kumar and Baker, 2000; Severing et al., 2012; Staiger and Brown, 2013; Szakonyi and Duque, 2018; Wang et al., 2018b).

## Upcoming Research and Conclusions

In the past few years, HTS technologies has unraveled the breadth of AS that is occurring in plants (Shen et al., 2014; Mandadi and Scholthof, 2015; Thatcher et al., 2016; Calixto et al., 2018; Zhang and Xiao, 2018). The availability of vast amounts of omics data (currently >1 Petabases), largely based on ShR RNA-seq and gene-level analysis, within the publicly available repositories such as the NCBI SRA database (Leinonen et al., 2011) offer new opportunities to data mine and uncover AS landscapes among diverse plants and conditions. Such studies will allow global determination of conserved AS landscapes, patterns, and phenomenon occurring among the diverse evolutionary lineages of plants. Furthermore, combining the ShR RNA-seq data with LoR RNA-seq will allow discovery of the low-abundant transcripts, and/or resolve inadequacies that exist in reconstructing transcripts and complex transcript structures. The HTS will also advance our knowledge of AS landscapes and processes in complex polyploid plant genomes, which has largely remained understudied when compared to diploid plant genomes. Lastly, despite being well-positioned to study AS in plants at an unprecedented scale using HTS technologies, the challenge ahead lies in deciphering the biological relevance and molecular function of the various alternatively spliced transcripts and the encoded proteins. Thus, we suggest that the AS research community place equal emphasis in the AS validation using reverse-genetic approaches.

## Author Contributions

RB, SI, EP, and KM designed the study and prepared the manuscript for submission. All authors have read and approved the manuscript.

### Conflict of Interest Statement

The authors declare that the research was conducted in the absence of any commercial or financial relationships that could be construed as a potential conflict of interest.
